# Health Alphabet

**Published:** 1884-08

**Authors:** 


### HEALTH ALPHABET.

The Ladies' Sanitary Association, of London, gives the following simple rules for keeping health, which we find copied in the *Sanitarian:*

A–s soon as you are up shake blanket and sheet;
B–etter be without shoes than sit with wet feet;
C–hildren, if healthy, are active, not still;
D–amp beds and damp clothes will both make you ill;
E–at slowly and always chew your food well;
F–reshen the air in the house where you dwell;
G–arments must never be made too tight;
H–omes should be healthy, airy, and light;
I–f you wish to be well, as you do I've no doubt,
J–ust open the windows before you go out;
K–eep the rooms always tidy and clean;
L–et dust on the furniture never be seen;
M–uch illness is caused by the want of pure air,
N–ow, to open the windows be ever your care;
O–ld rags and old rubbish should never be kept;
P–eople should see that their floors are well swept;
Q–uick movements in children are healthy and right;
R–emember the young cannot thrive without light;
S–ee that the cistern is clean to the brim;
T–ake care that your dress is all tidy and trim;
U–se your nose to find if there be a bad drain;
V–ery sad are the fevers that come in its train;
W–alk as much as you can without feeling fatigue;
X–erxes could walk full many a league.
Y–our health is your wealth, which your wisdom must keep;
Z–eal will help a good cause, and the good you will reap.

